# Minimal Information Data-Modelling (MID) and an Easily Implementable Low-Cost SHM System for Use on a Short-Span Bridge

**DOI:** 10.3390/s23146328

**Published:** 2023-07-12

**Authors:** Connor O’Higgins, David Hester, Patrick McGetrick, Elizabeth J. Cross, Wai Kei Ao, James Brownjohn

**Affiliations:** 1 School of Natural and Built Environment, Queen’s University Belfast, University Road, Belfast BT7 1NN, UK; 2 School of Engineering, University of Galway, University Road, H91 TK33 Galway, Ireland; 3 Department of Mechanical Engineering, University of Sheffield, Mappin Street, Sheffield S1 3JD, UK; 4 Department of Civil and Environmental Engineering, The Hong Kong Polytechnic University, Hong Kong; 5 Vibration Engineering Section, College of Engineering, Mathematics and Physical Sciences, The University of Exeter, Exeter EX4 4QF, UK

**Keywords:** structural health monitoring, data modelling, environmental effects, low-cost, long-term bridge monitoring, regression

## Abstract

Structural Health Monitoring (SHM) is a technique that involves gathering information to ensure that a structure is safe and behaving as expected. Within SHM, vibration-based monitoring is generally seen as one of the more cost-effective types of monitoring. However, vibration-based monitoring has mostly been undertaken on long-span bridges using data collected with a dense network of sensors. Historically, the logistical difficulty of collecting data on short- and medium-span bridges has meant that the usefulness of vibration-based methods on these bridges is largely unknown. Therefore, this study proposes Minimal Information Data-modelling (MID). MID is an approach that utilises low-cost, easily implementable sensors that are potentially feasible for operators to purchase and operate across a network. This approach will be investigated to determine whether MID is a feasible approach for monitoring short- and medium- span bridges. The results from MID were assessed to determine whether they could detect a suitably small shift in frequency, which is indicative of damage. It was determined that the data models could reliably detect frequency shifts as low as 0.01 Hz. This magnitude of frequency shift is similar to the level of frequency shift reported for a range of bridge damage cases found by others and validated with FE models. The accuracy achieved by the data models indicates that MID could potentially be used as a damage detection method. The cost of the equipment used to collect the data was approximately £370, demonstrating that it is feasible to use MID to monitor bridges across an entire network.

## 1. Introduction

This work introduces Minimal Information Data-modelling (MID), a novel Structural Health Monitoring (SHM) approach that addresses the limitations of conventional SHM systems, particularly in the context of short- and medium-span bridges. The paper highlights the potential significance of implementing SHM systems across an entire bridge network, emphasizing the need for low-cost instrumentation that is economically feasible to install. By presenting MID as a cost-effective alternative, the paper contributes to the literature by filling the gap in SHM research focused on small- to medium-sized bridges.

When it comes to undertaking SHM on bridges, most studies have focused on long-span bridges. This is evident from the numerous studies that have installed instrumentation on bridges over 150 m in length [1,2,3,4,5,6]. It is common for civil structures to have large numbers of sensors installed for structural health monitoring (SHM). In fact, [7] noted that it was not uncommon for a single civil structure to have hundreds or even thousands of sensors. This tendency is again evident in previous studies, with [3] installing 232 sensors on a 1.1 km bridge, [4] installing more than 600 sensors on a tower, and [5] installing 114 sensors on a 168 m bridge. While some SHM has been done on shorter bridges, such as by [8,9,10,11], these structures still tend to use large numbers of sensors, ranging from 40 to over 200. However, using a large number of sensors is not practical for an entire bridge network due to logistical and financial constraints. Although some studies have explored the use of low-cost sensors for SHM, they have generally been limited to laboratory settings [12] or short-term monitoring, as in [13] (30 min) and [14] (30 days).

The focus of this paper is starting with an SHM system that is economically feasible to install across a network of bridges. To that end, the SHM system must have three properties: it must (1) be low cost, (2) be easily installed, and (3) provide useful information about the behaviour of each bridge. There is a range of SHM methods that can be used to monitor bridges.

Studies show that strain-based Structural Health Monitoring (SHM) systems are effective in evaluating bridges. These systems can identify issues such as cracking or excessive loading and provide valuable information to bridge managers. Examples include installing strain sensors on a Warren truss girder bridge to increase design life and determine traffic characteristics [15] and using strain sensors on a prestressed concrete continuous box-girder bridge to find unknown properties and provide a reliability assessment [16]. Bridge weigh-in-motion systems have also been successfully employed using strain sensors to provide crucial information on changing traffic loads over time [17,18]. While the strain is a very interpretable way of collecting measurements from a bridge, stain sensors generally only measure local effects, and so, require multi sensors systems to get a global picture of the bridge.

Computer vision techniques uses pattern-matching algorithms to compare multiple images of an object and detect changes. This method can detect properties such as corrosion, cracking, and displacement in structures like bridges. Vision-based monitoring systems do not require sensor installation and can measure both local and global effects. Some studies use a feature-based approach that tracks salient features to eliminate the need for target placement [19,20]. This approach shows promise in situations where traditional SHM systems may be cost-prohibitive, but these methods have generally not been used to collect long-term data.

Vibration-based monitoring is a cost-effective way to monitor the health of a structure that satisfies both low-cost and easy installation criteria. As the vibration of the structure is a global property, it can be measured at different locations on the bridge, making installation easier. The monitoring equipment can be installed on the top surface without disrupting traffic. However, other sensors such as strain are usually placed on the underside of the deck, requiring specialized access equipment or road closures. Changes in a bridge’s natural frequencies, damping ratio, and mode shapes can indicate damage. However, variations in the environment and operations can also affect these properties; this has been shown in many studies. For instance, [10,21,22,23] showed that these normal variations would typically mask the change in frequency caused by the presence of damage.

While vibration-based monitoring and data modelling have been successful in long-span bridges, such as in [24], with dense sensor networks, few studies have focused on short- and medium-span bridges. Vibration has been shown to aid in the production and updating of digital twin models on medium-span bridges [25]. However, in general, the absence of studies on the level of information that could be provided to bridge managers from a data model trained on a minimal amount of data from a low-cost SHM system has made low-cost SHM challenging to implement in practice, especially when detecting and locating damage.

One of the main focuses of this work is to determine whether MID can provide useful information, which is a challenging criterion to prove. To establish a benchmark level of performance required, a review of other damage detection studies was conducted. The studies proposed a vibration-based method and then tested the damage detection capabilities of the method using either numerical simulation, experimentally using scale models, or both. Although the damage simulated in these studies varied, it could generally be represented by a localised loss in stiffness. Studies that used only numerical models [17,18,19,20,21,22,23] were able to detect damage ranging from a 5% to 66% loss of stiffness, while those that used a combination of numerical and experimental studies [19,24,25,26,27,28,29,30] were able to detect damage ranging from a 7% to 49% loss of stiffness. The range of detectable damage for bridge SHM systems is from 5% to 66% loss of stiffness, with significant variation in both the longitudinal length and transverse width over which the damage is implemented. However, a localised stiffness loss of 25% is representative of what is being claimed as detectable in studies with limited or no confounding effects. Therefore, a 25% localised loss in stiffness is the target for this paper when assessing whether MID can provide useful information.

The paper’s specific contributions can be summarized as follows:

1. The development of a data modelling workflow suitable for sparsely instrumented bridges: The paper presents a data modelling workflow specifically designed for bridges with minimal instrumentation. This addresses the gap in research by providing a methodology that accommodates the sparse sensing data envisaged by MID.
2. The detection of abnormal frequency shifts and comparable damage levels: The paper demonstrates MID’s ability to detect abnormal frequency shifts, comparable to or smaller than those reported in previous studies that observed frequency shifts due to damage.

Testing the MID data models reveals the system’s sensitivity to frequency shifts/variations as small as 0.01 Hz. This finding suggests that MID holds potential as a damage detection method. Additionally, the low cost of the equipment used in data collection (£370) demonstrates the feasibility of employing MID to monitor bridges across a network. This cost is for the individual sensors used in this study; the actual cost will vary depending on the quality of the utilised sensor and the number of sensors, but our cost approximation does give an indication of what can be gained using a SHM system at this cost level. The capabilities of MID, combined with its cost-effectiveness and ease of installation, hold promise for the widespread implementation of SHM systems, thereby potentially enhancing the safety and management of bridge networks.

## 2. Description of Bridge and Monitoring System

**Figure 1:**
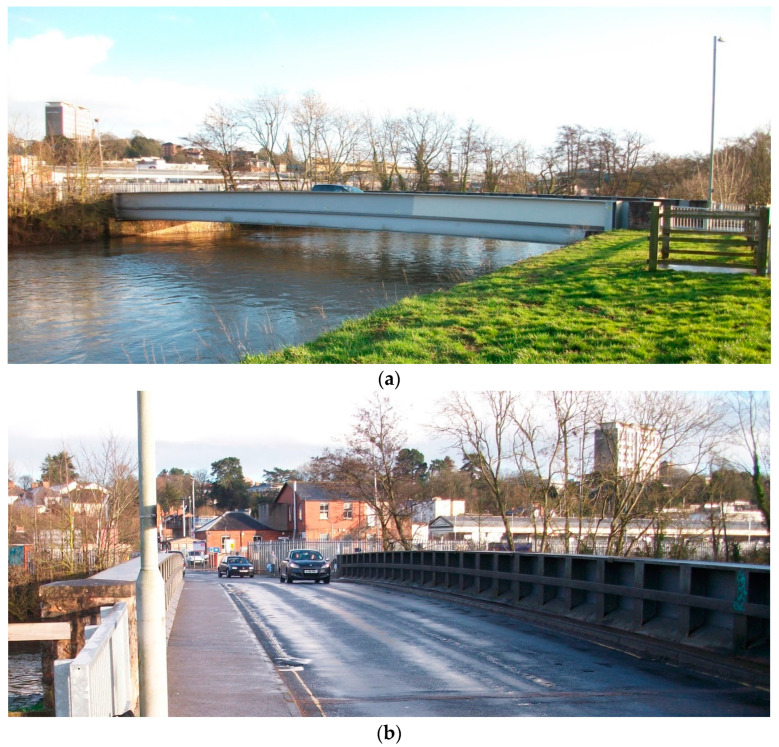
Bridge used in study: (**a**) north elevation; (**b**) bridge deck looking to the east.

The bridge monitored in this study is a half-through steel girder bridge with a 36 m span and simply supported deck (Figure 1). The bridge carries two lanes of traffic and one pedestrian footpath located on the north of the bridge. The bridge is in an urban area that experiences mostly cars, but also a limited number of HGVs. The 7.6 m wide, 200 mm deep concrete deck is supported on a series of 450 mm deep steel beams spanning transversely between the main girders, which are approximately 2 m deep. The interior of the main girders also includes a series of stiffeners, which run for the length of the bridge.

### 2.1. Modal Test

**Figure 2:**
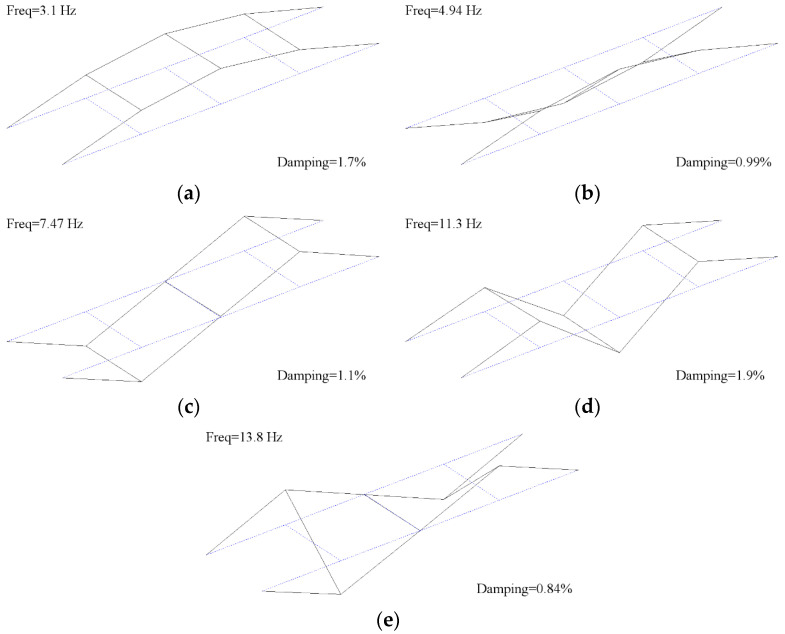
Bridge mode shapes and frequencies (**a**) Mode 1: First bending mode (**b**) Mode 2: First torsion mode (**c**) Mode 3: Second bending mode (**d**) Mode 4: Third bending mode (**e**) Mode 5: Second torsion mode.

The modal testing of the bridge is outlined in [26]. The acceleration data for the modal test was collected using force balance accelerometers and analysed using the NExT/ERA operational modal analysis (OMA) procedure. The test identified five modes, ranging from 3.1 Hz to 13.8 Hz, whose mode shapes and associated frequencies are presented in Figure 2.

### 2.2. Description of Long-Term Monitoring System

**Figure 3:**
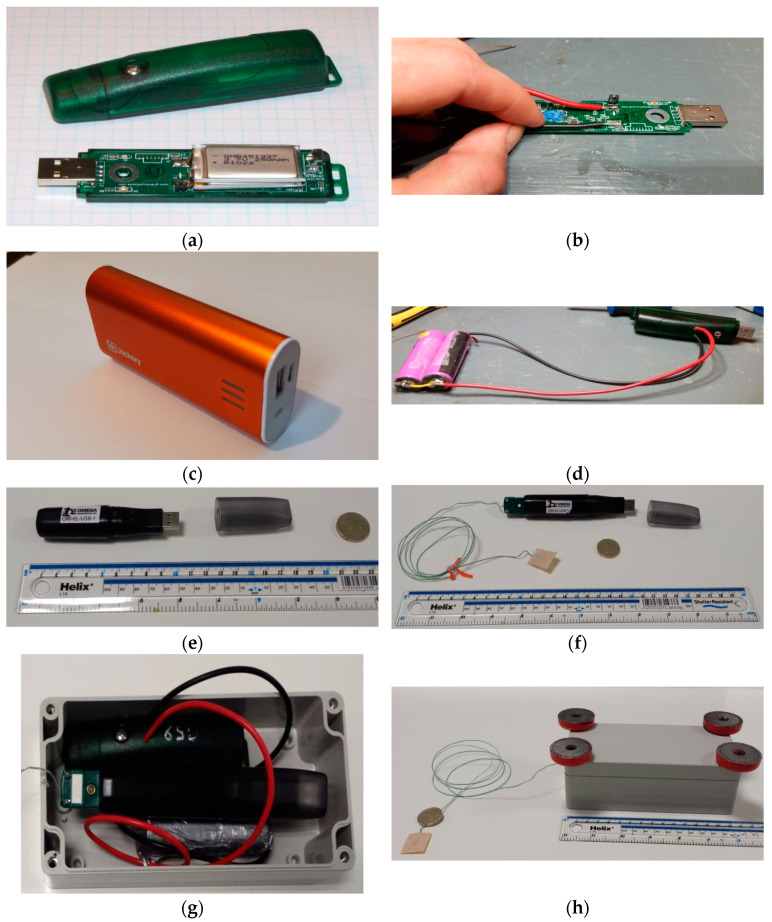
Long-term monitoring system: (**a**) original GCDC accelerometer, (**b**) removing accelerometer battery and soldering on wires for connection to a larger battery, (**c**) battery booster pack used, (**d**) completed accelerometer alterations, (**e**) air temperature sensor, (**f**) surface temperature sensor, (**g**) accelerometer and surface temperature sensor in enclosure, and (**h**) completed enclosure with magnets fitted.

Sensor cost and ease of installation were the primary motivations when choosing the SHM system whose implementation mirrored constraints faced by real-world applications. The chosen SHM system consisted of one MEMS accelerometer and two temperature sensors with a total cost of approximately £370. Acceleration, unlike some bridge responses such as strain, is affected by damage anywhere in the structure, not just close to the sensor’s location. The accelerometer used was the ‘USB Accelerometer X2-2′ from Gulf Coast Data Concepts (see Figure 3a). This accelerometer measures acceleration in three axes within a range of ±2 g and has a real-time clock to timestamp every acceleration measurement, with data stored locally on an SD card at a sample rate of 128 Hz. The accelerometer is powered by an internal battery with standard life that allows for 10 h of data collection. For this system, the standard battery was replaced with a wired connection (Figure 3b), which allowed the sensor to be powered by a larger external battery pack (Figure 3c) that allowed data collection for 20 days before needing a recharge. Since this deployment, X2 accelerometers have been improved with longer battery life [27], and so modifications would no longer be required.

The temperature sensors used were the ‘OM-EL-USB Series’ from Omega Engineering [28]. Two sensors were used: one to measure the air temperature (Figure 3e) and one to measure the surface temperature at a point on one of the main steel beams. For the surface temperature readings, a self-adhesive thermocouple patch (Figure 3f) was attached to the steel surface. These temperature sensors have a resolution of 0.5 °C and, like the accelerometers, store data locally with corresponding timestamps.

A monitoring enclosure (Figure 3g) was used to house the accelerometer and surface temperature sensor. The enclosure provided both security and weatherproofing for the sensors. Attaching magnets to the top of the enclosure (Figure 3h) allowed it to be fixed to the underside of the primary beam’s top flange without the need for mechanical fixings.

As a single accelerometer was used to collect the long-term vibration data, its position was an important consideration. The mode shapes obtained from the modal test informed this decision. Positioning a sensor close to the bridge’s quarter-span point allows the sensor to detect the greatest number of modes/frequencies. The air temperature sensor (Figure 3e) was not placed within the enclosure as solar gain inside the enclosure would affect the temperature measurement. Instead, the temperature sensor was placed on the bearing shelf on the north side of the east abutment. This location was out of direct sunlight, giving a representative value of local air temperature.

**Figure 4:**
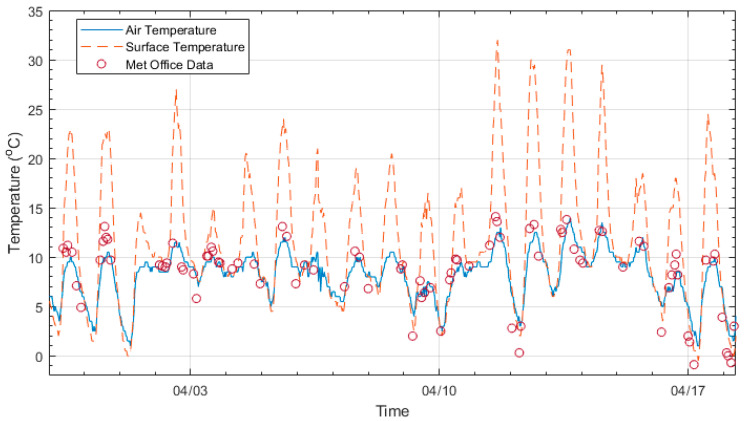
Twenty days of air temperature and surface temperature data recorded at the bridge site and air temperature data recorded at a nearby weather station.

As described above, temperature data was collected from the surface of the main steel beam and the air temperature (measured at the abutment). Figure 4 shows 20 days of temperature data with the solid and dashed lines representing the air temperature and surface temperature, respectively. Both air and surface temperature show the same daily trends. However, the surface temperature shows significantly higher maximum values. The higher surface temperature readings are believed to be due to the solar gain of the steel girder’s top flange.

To check the air temperature sensor’s accuracy, the corresponding air temperature has also been plotted for a nearby weather station (circular data markers). A Met Office weather station approximately 5 miles from the bridge collected the data. There is good agreement between the air temperature collected at the bridge site and that collected from the weather station. This suggests that the air temperatures readings collected from the SHM system are credible.

## 3. Finite Element Analysis

### 3.1. Finite Element Analysis Description

Finite Element Analysis (FEA) has emerged as a powerful tool in civil engineering for the analysis and design of complex structures. FEA has also been shown to a useful tool in the management of bridges [8,29]. FEA is based on the principles of numerical approximation and the subdivision of a structure into smaller manageable elements. FEA can accurately predict structural behaviour under various loading conditions, boundary conditions, and material properties. FEA involves the discretization of a structure into finite elements representing small regions within the structure. Each element is characterized by a set of mathematical equations, which are solved iteratively to determine the response of the structure. By assembling these elements, the complete structural behaviour can be determined. A more detailed description of FEA can be found in [30].

FEA provides a robust framework for simulating the behaviour of bridge structures under a wide range of loading scenarios, boundary conditions, and material properties. Through FEA, the structural response to static, dynamic, and environmental loads can be accurately predicted. FEA also offers a valuable means of validating monitoring data [31,32] and identifying potential damage [33] or deterioration in bridge structures. By comparing the FEA predictions with measured data from sensors installed on a bridge, engineers can assess the accuracy of the model and detect any discrepancies. If significant deviations are observed, it may indicate the presence of damage or deterioration within the structure.

Despite its numerous advantages, FEA is not without limitations and uncertainties. The accuracy of FEA results depends on the quality of input data, including material properties, boundary conditions, and geometric modelling. Assumptions made in the modelling process, such as linear material behaviour and the neglecting of certain non-linearities, may introduce uncertainties in the predictions. Moreover, FEA involves discretization and meshing processes, which introduce discretization errors. The convergence of the iterative solution process also poses challenges as inappropriate mesh densities or solution settings may yield inaccurate results. Furthermore, the complexity of modelling bridge–structure interactions, such as expansion joints, bearings, and soil–structure interaction, adds additional sources of uncertainty.

### 3.2. Developing Finite Element Models of the Bridge

This section will describe the creation of the FE model using the construction drawings and other available resources. The section will also describe the approach adopted for updating the model using the modal test data to simulate accurate natural frequencies. Lusas version 19 was used to create the FE models described in this study, starting with the geometry of the bridge obtained from a combination of construction drawings and on-site measurements.

**Figure 5:**
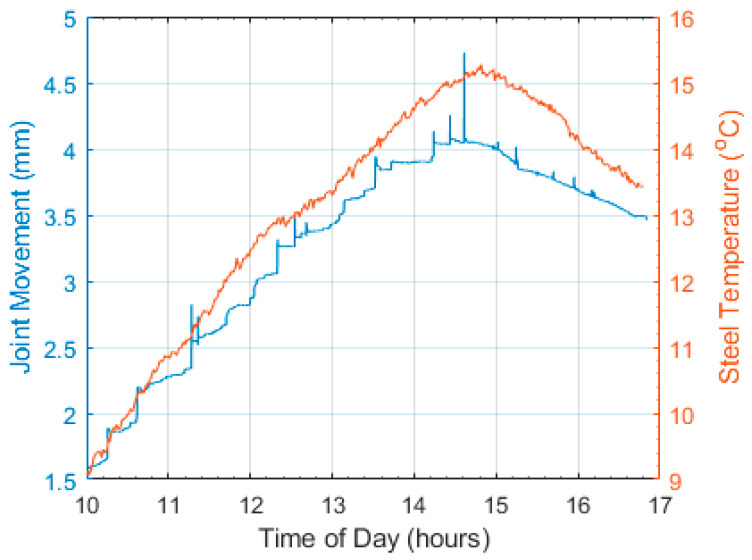
Bearing movements and temperature variation observed during monitoring.

On the day of the modal test on the bridge, the bearing movement of the roller end of the bridge was measured using an LVDT and the results of the monitoring are shown in Figure 5, with displacement and temperature plotted on the left and right y-axis, respectively. It can be seen in the figure that while the bearing movement is approximately proportional to temperature, the movement of the bearing is not smooth; instead, it exhibits a stick–slip pattern. Thus, while the bearing is a roller in ambient conditions, it is behaving more like a pin–pin, and this is what was simulated in the FE model. More information about this monitoring can be found in [34].

Table 1 (below) shows the material properties for concrete and steel that were used for the initial FE model prior to undertaking the updating procedure.

**Figure 6:**
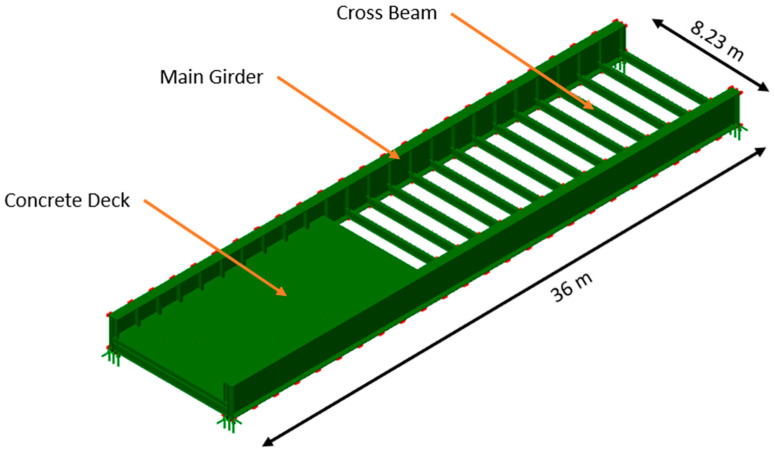
Main structural elements of the bridge and key dimensions.

The main geometric features of the FE model are given below and can also be seen in Figure 6. In this figure, the main structural elements of the bridges have been annotated and the key dimensions marked for reference.

- Total length of bridge/main girders: 36 m
- Total width of bridge/cross girder length: 8.23 m
- Number of cross members/distance between members: 25 no./1.5 m
- Depth of main girders: 2.1 m

After the initial model was created in the software, the FE model was updated to obtain the best match of nature frequencies with modal test values. Only one variable was chosen to be updated to avoid the complex interaction caused when two of the material properties are varied simultaneously; hence, density of the concrete deck was chosen as the variable to be updated. While a thin layer of surfacing is indicated on the drawings (from 1975), the depth of surfacing is not clear. The surfacing was assumed not to contribute to the deck’s stiffness, but rather, to add mass, and so, slightly increasing the density of the concrete would capture the mass of the surfacing layer. The surfacing layer is the greatest uncertainty due to the information gathered about the other parameters.

To update the FE model, an automated program was written to vary the concrete deck density in increments of 5 kg/m^3^ and extract the resulting natural frequencies. The percentage difference between the modal test frequencies and the FE frequencies was then used to select the density that resulted in the most accurate frequencies.

After updating the model, the best fit (to the modal frequencies) for the density of the concrete deck was set at 2625 kg/m^3^. Table 2 shows the corresponding natural frequencies compared to the values from modal analysis, together with differences in Hz and %, showing differences of less than 2.5% for three of the modes. For mode 2 the difference was 7.89% indicating that the model was not quite capturing the behaviour of this mode correctly. This was not unexpected as the relatively simple updating procedure, assuming pinned—pinned and updating the deck density, was likely to struggle to perfectly capture the individual behaviour for all modes. However, overall, the frequency predictions of the model agreed with the measured values across multiple modes, indicating that the model was an accurate representation of the bridge. Therefore, the frequency predicted by the model when damage was simulated was also likely to be accurate.

The FE model developed in this section will be used in Section 7 to simulate the occurrence of damage to investigate the capabilities of the data model developed in Section 5 of this paper.

## 4. Extraction of Frequency Data and Analysis of Environmental Effects

This section presents an overview of the bridge natural frequency data collected, which will later be used in the data model (Section 5). Reviewing the frequency data ensures that the data is creditable and allows trends and correlations between the frequency data and temperature to be observed. This step ensures that the best methods for the data modelling process are used as different relationships between the variables will require different modelling techniques.

Numerous previous studies [10,35,36] have proposed tracking the natural frequency of a structure as an indicator of its health. A Stochastic Subspace Identification (SSI) method was used to convert acceleration time-series data to frequency data. SSI is a commonly used procedure for frequency extraction and has been applied to acceleration data in many long-term monitoring studies such as [9,37,38]. Specific details have been well explained by others previously—for e.g., [39] provides a comprehensive review including the necessary steps and the differences between variants of the SSI procedure. In this study, the acceleration data was divided into 30-min sections and the natural frequency was extracted from each of these 30-min windows of data.

**Figure 7:**
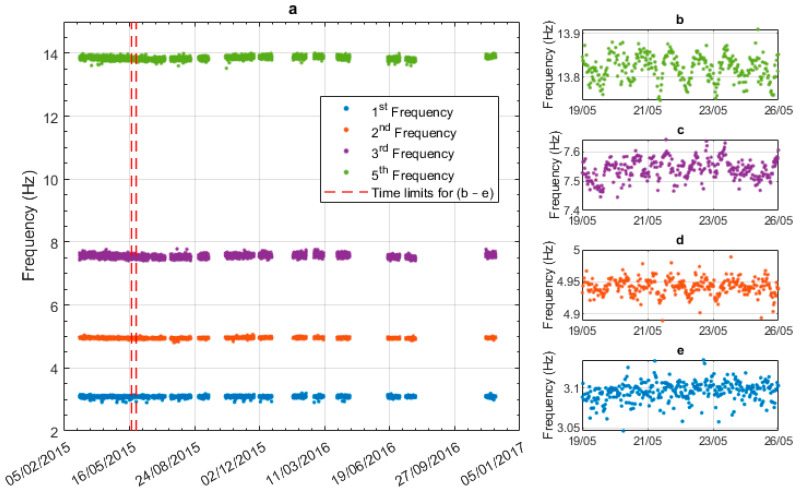
Modal frequencies extracted from consecutive 30-min windows using SSI: (**a**) all frequencies over the entire monitoring period, (**b**) mode 5 frequency over 7 days, (**c**) mode 3 frequency over 7 days, (**d**) mode 2 frequency over 7 days, and (**e**) mode 1 frequency over 7 days.

Figure 7a shows all frequencies over the entire monitoring period, from February 2015 to November 2016, with the vertical red dashed lines showing the time limits for plots (b) to (e). Figure 7b shows seven days of data for the 5th natural frequency, and Figure 7c–e shows the same period for modes 3 to 1. The position of the sensor was close to the node point of mode 4, and hence, mode-4 vibrations were not captured. Figure 7b shows a clear daily frequency cycle in the 5th mode. The cycle is less apparent in the 3rd and 2nd modes and is not really evident in the 1st mode. Due to the scale in Figure 7a the annual natural frequency trends are not evident; these will be discussed later in this section.

Table 3 gives the mean, range, and relative variation percentage for each of the four modes. The relative variation is defined by Equation 1, and the whole monitoring period is used to calculate the relative variation. To calculate the range of the natural frequency measurements, a basic outlier detection method was used so that outliers would not skew the results. The outlier method used served to remove observations that were more than three standard deviations away from the mean. The relative variation metric has been used in previous studies such as [40], and it allows the frequency range to be expressed as a percentage of the mean frequency of the mode.

(1) $${RV}_{i}=\frac{f_{max}-f_{min}}{f_{mean}}\times 100$$

Here, $f_{max}$, $f_{min}$, and $f_{mean}$ are the maximum, minimum, and mean of the measured frequencies for a specific mode (i), respectively.

The relative variation of the measured frequencies ranges from 1.96% to 4.30%. This variation is caused by the changing environmental and operational effects, and would likely mask the presence of damage, if damage were to occur. The goal of the data model developed in Section 5 is to remove this variation so that frequency shifts due to damage can be more easily detected.

Prior to creating the data models, the relationships between frequency and temperature should be examined. This serves two purposes:

1. To confirm that the bridge behaves broadly in line with expected patterns to give confidence in frequency measurements. For e.g., for a typical bridge, it would be expected that the natural frequency is inversely correlated with the temperature.
2. To determine which modes have the most significant correlations with temperature.

**Figure 8:**
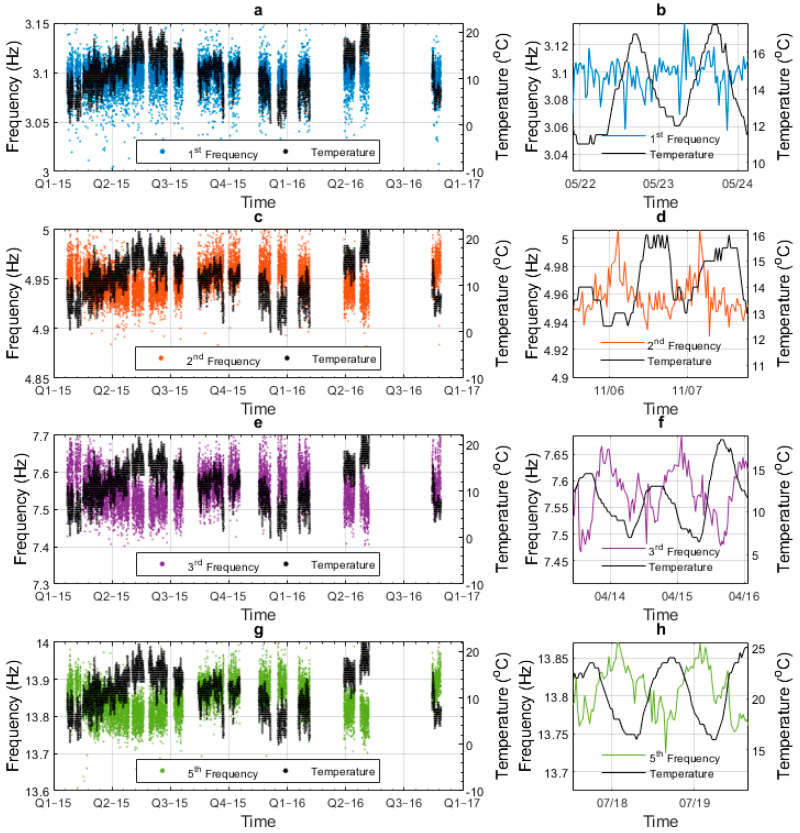
Air temperature and frequency data for four different modes: (**a**) mode-1 frequency and air temperature data for full monitoring period, (**b**) mode-1 frequency and air temperature data for two days, (**c**) mode-2 frequency and air temperature data for full monitoring period, (**d**) mode-2 frequency and air temperature data for two days, (**e**) mode-3 frequency and air temperature data for full monitoring period, (**f**) mode-3 frequency and air temperature data for two days, (**g**) mode-5 frequency and air temperature data for full monitoring period, and (**h**) mode-5 frequency and air temperature data for two days.

Figure 8 plots the identified natural frequency and temperature data together against time. The blue dots in Figure 8a represent the mode 1 frequency and the black dots (plotted with respect to the right-hand-side y-axis) show the air temperature at the time the frequency measurement was observed. The data covers the period from early 2015 to late 2016, albeit with gaps due to a lack of available personnel to collect the data and change batteries. For the duration of the monitoring period, the annual temperature cycle is evident, but the mode-1 frequency remains relatively stable at around 3.1 Hz. Therefore, we can conclude that for this range of temperature, the mode-1 frequency is relatively insensitive to annual temperature variation. Figure 8b shows the mode-1 frequency and temperature data observed over two days to illustrate daily trends. The mode-1 frequency again appears relatively insensitive to temperature when viewed on a daily cycle. Rows 2 to 4 of the figure show the same information for modes 2, 3, and 5, respectively (see the mode shapes and frequencies shown previously in Figure 2). Unlike for mode 1, the frequencies of these modes appear to have negative correlations with temperature, which is consistent with the findings of other studies. No data is available for mode 4 (third bending mode, 11.3 Hz), with the reason for this being that the node for this mode is approximately at the 1/3 point of the span (see Figure 2d) and so the sensor (located at the 1/4 span) captures little energy from this mode.

## 5. Data Modelling for Use with Sparse Data

The general premise for data modelling like the kind used herein is as follows: the environmental observations from the bridge are used as the inputs to the data model, and the purpose of the data model is to predict how the natural frequencies change based on the changing environmental measurements. If the data model does a sufficiently good job of predicting how the environment affects the natural frequencies, then these variations can be removed from the data, leaving a residual that, in theory, should be free from environmental effects and, therefore, a lot more sensitive to other effects such as damage. In practice, these environmental effects cannot be completely removed, but if a large enough proportion of the environmental effects are removed, then it may be possible to detect damage.

In Section 1, the need for a customised data modelling method/workflow suitable for applications with sparsely instrumented short- and medium-span bridges was identified. Consequently, this section aims to develop such a workflow and identify what information on frequency shifts could be returned to the bridge manager using such a data model. Section 5.1 identifies suitable modelling methods. In Section 5.2 a data modelling workflow is presented that is suitable for modelling the kind of bridge data that was obtained from the sparse sensor arrangement envisaged by the MID approach. Section 5.2 also describes how the workflow was applied to the bridge data presented in Section 4, and finally, Section 5.3 presents the results from the data model.

### 5.1. Identifying Suitable Methods

As MID is intended to function with minimal data, it was decided to use regression techniques for the data modelling, as regression methods can add a level of complexity to compensate for the small number of variables. With this in mind, linear regression and Gaussian process regression (GPR) methods will form the base of the data models in the typical workflow. Linear regression is an appropriate method as some of the relationships between variables were observed to be linear; this will be discussed in more detail later. Any other relationships between the variables can be modelled using GPR as this method offers a universal approximator, works for low numbers of data points, and is Bayesian. As it is a Bayesian method, a probability distribution is obtained rather than a single point prediction. The resulting confidence intervals can also be beneficial, particularly when operating with limited data. Having selected the broad data modelling approach, the next step is to develop the specific workflow to be used. Section 5.2 describes the data modelling workflow proposed for MID. The various subtle steps/checks described in the workflow are crucial for MID; when dealing with limited data, it is imperative that it is appropriately analysed to exploit the data to its full potential.

### 5.2. Workflow Developed for Data Modelling

#### 5.2.1. Overview of Approach

**Figure 9:**
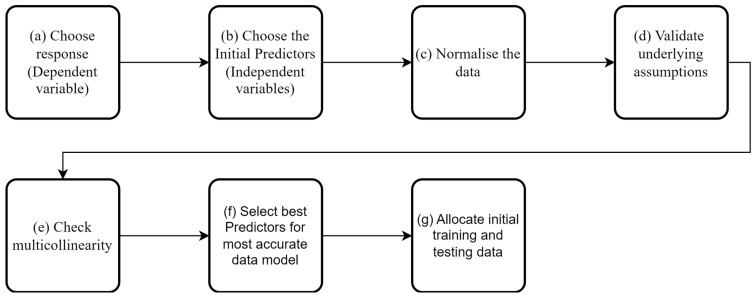
Flow chart showing the workflow for MID.

Many studies have used data modelling for SHM [35,41,42,43]. However, a workflow for modelling sparse data collected from short- and medium-span bridges has not been presented in the current SHM literature. While broad principles of data modelling are known, each data set is subtly different, hence the need to identify/develop a workflow that will work for the spare sensing data envisaged by MID. The data models utilised in other studies have also been trained with large data sets and, as such, will differ in approach from the data models used here. This section presents the developed workflow and considerations that should be made when using a limited dataset in conjunction with a data model and, in particular, a regression analysis. Figure 9 presents a flow chart of the steps involved, and the bullet points below give a brief synopsis of the individual steps.

As shown above, when creating a data model using regression techniques, several factors need to be considered. Two of the most critical factors are the selections of the dependant and independent variables, which will be covered in more detail in Section 5.2.2. This section will give a brief overview of the other factors that should be considered before training the data models.

(a) **Choose dependent variable**: This is the variable of interest: in this case, the natural frequency of the bridge; for e.g., the frequency of the fifth mode. This is discussed in more detail in Section 5.2.2.
(b) **Choose independent variables**: These variables are used to predict the dependent variable. The choice of these variables is discussed further in Section 5.2.2.
(c) **Normalisation of the data**: While not all regression methods require the data to be normalised, linear regression methods are affected by non-normalised data. The normalisation of the data can also be necessary when determining which of the predictors is the most influential in the data models. In this study, the dependent variable and all independent variables have been normalised.
(d) **Validate Underlying Assumptions**: Before any regression analysis, the following assumptions for the data need to be validated:
  - The linearity of the phenomenon measured (for linear regression);
  - The constant variance of the error terms;
  - The independence of the error terms; and
  - The normality of the error term distribution.

The testing of assumptions i to iii is usually done by plotting residuals against predicted responses. The distribution of the resulting graph allows the checking of any assumption violations. The details of how to check the assumptions can be found in [44]. The last assumption (iv) is the normality of the error term distribution, which is checked by plotting the residual distribution.

(e) **Multicollinearity**: Multicollinearity occurs when independent variables in a regression model are correlated with each other. If this happens, it is hard to isolate the relationship between each of the independent variables and the dependent variables. Multicollinearity will generally weaken the predictive power of a regression model. Multicollinearity is typically more of an issue when many variables are being studied. In this paper, there are relatively few variables. However, six of the variables are temperature-based (discussed in Section 5.2.2). The likelihood that these variables will be correlated is high, and so it is essential to ensure they will not adversely affect the data model. Several methods can be used to detect multicollinearity; the method used here is the one proposed by [45]. This method uses the condition index and the regression coefficient variance to determine the overall level of multicollinearity. When checked, the surface temperature and the surface temperature 1 h prior variables exceeded the multicollinearity threshold, and so surface temperature 1 h prior was excluded from the study.
(f) **Select best predictors**: Removing independent variables with little predicting power is important for several reasons:
  - It reduces the time needed to train the regression.
  - It reduces the complexity of the regression analysis.
  - It allows the analyst to gain a better understanding of the behaviour of the structure. Filtering out the less important factors identifies which of the remaining factors are most strongly correlated with the dependent variable’s observed behaviour.

There are many different methods available to aid feature selection in a regression context. Here, a relief-based approach is adopted; relief-based approaches avoid the need to carry out an exhaustive comparison between candidate features, instead relying on a nearest-neighbour-type measure to assess inter-relationships. ReliefF [46] is a variant that assigns weights to the predictors given a set of observations. A higher weighting indicates the predictor’s relevance to the dependent variable (frequency in this case). A comprehensive review and details on the implementation of ReliefF can be found in [47]. Using the ReliefF method determined that all the remaining predictors positively affected the data model.

(g) **Allocate initial training and testing data**: The next step is to decide how much data to use for training of the model and how much to use for testing of the model. To have the best predictive power the model will most likely need to be trained with ‘one cycle of data’. This data should include all expected ranges of temperatures. Here, the initial choice of training data was March (year 1) to April (year 2), spanning one cycle of the annual environmental variations. The remaining data, spanning from June (year 2) to Dec (year 2), was used to test the model after it had been trained.

All the created data models use validation within the training phase of the data model. Retaining a portion of the training data (holdout validation) to validate models helps to prevent the overfitting of the models [44].

Once the above steps are complete, the first iteration of the data model is complete. It is also necessary to check how outliers in the data set affect the model, and the process for doing this is described in more detail in Section 5.2.3.

#### 5.2.2. Choose Dependant and Independent Variables

The response or dependent variable could be any of the extracted frequencies, i.e., the frequencies of modes 1, 2, 3, or 5. If all four of the extracted frequencies were to be studied, then, typically, four different data models would be needed. However, to avoid repetition when describing the workflow, only the 5th frequency is reported in detail (i.e., it is selected as the dependent variable). This mode showed significant variation with temperature and, consequently, the greatest need for removing variation due to environmental effects. However, the same process can be applied to any of the bridge’s natural frequencies.

When using MID, a balance needs to be struck between having predictors that sufficiently influence the dependent variable while also having the predictors be easy to measure with low-cost equipment. In previous studies, environmental factors have been shown to have the most significant influence on natural frequencies.

The nine predictors that were chosen for the base data models and the reasons for choosing them are as follows:

(a) Air temperature and surface temperature at the time of frequency measurement (2 predictors).
(b) Air temperature and surface temperature 1, 6, and 12 h prior to frequency measurement to capture any potential thermal lag of the material/bridge structure (6 predictors).
(c) Damping ratio estimate obtained from the SSI method (1 predictor).

(a) and (b), both being temperature-based variables, have been used in previous studies [1,48] to model natural frequency variations. (c) has been shown to vary with natural frequency in these previous studies [1,49]. All predictors were included by default in our data model. However, some may have been excluded when testing for problems such as multicollinearity.

Two accelerometers were used in alternate duty: one with batteries being charged and data downloaded and the other to collect data. To avoid possible differences due to using different sensors, it was decided not to use metrics such as Root Mean Squared (RMS) acceleration as predictors. In future studies, the accelerometer will remain in place with constant power provision, and so it will be possible to use raw acceleration data and/or metrics from the raw acceleration data, such as RMS values, as predictors.

#### 5.2.3. Outlier Detection and Removal

In this study it was determined that the removal of outliers led to a marginal improvement in the regression models, and a likely cause of the outliers was identified. Outliers were found to occur at times of low signal-to-noise ratio (SNR), for e.g., at nighttime when there was little or no traffic in a particular 30 min window of data. A low SNR challenges all OMA procedures, reducing estimation reliability [50].

Outliers in the dataset can affect the results of the regression depending on how they differ from the mean of the data. Outliers will affect the fit of the regression as the model may be pulled towards the outlying points to the detriment of the overall fit. The inclusion of many outliers in the training data would change the apparent ‘normal’ behaviour. When this data is compared with new data which includes truly anomalous data points, these outliers may not be sufficiently different to flag a problem.

Decisions on whether to remove/retain a potential outlier from the dataset can significantly impact the final model, and so, this needs to be approached systematically. The framework used for decisions on the removal of outliers in this study is outlined in [51].

**Figure 10:**
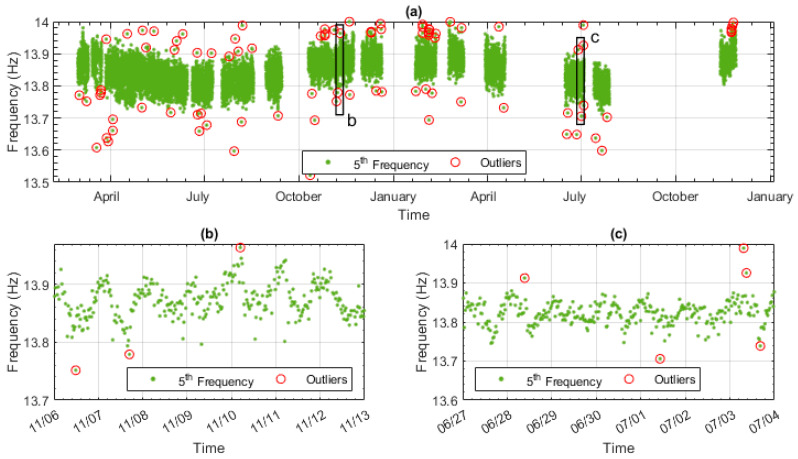
Outlier detection results over (**a**) the whole monitoring period, (**b**) week of training data, shown in (**a**) by frame labelled b, and (**c**) week of testing data, shown in (**a**) by frame labelled c.

Many outlier detection methods [52] have been derived from disciplines including econometrics and big data analytics. The method chosen in this study was moving Median Absolute Deviation (MAD), based on a previous study [51] which found that the windowing approach, such as that used in the moving MAD method, is suited to data with a varying mean such as natural frequency data. After applying the moving MAD method, the points that are considered outliers in the frequency data are shown in Figure 10. Figure 10a shows the outliers (red circles) that are detected in the whole monitoring period. Figure 10b,c show a smaller proportion of the data—namely, a week’s worth of data from both the training and testing data.

This study of the outliers found that the average time at which the outliers occurred was 09:00 in the morning, while the average time of the dataset as a whole was 12:10 in the afternoon. This discrepancy prompted an investigation of when the outliers occurred, and it was determined that 63% of them occurred between the hours of 7 pm and 6 am. During this period, the traffic is generally below average. The most likely explanation is the reduction in reliability of natural frequency estimation with a low SNR. As the exclusion of the outliers has been shown to improve the data model and the data points identified as outliers are shown to be caused by a low SNR, they can be removed from the data set.

### 5.3. Data Model Results

This section describes the choice of metrics used to compare the performance of the four data models (one model for each of the modes 1, 2, 3, and 5). The root mean square error (RMSE) is the square root of the residuals’ variance and represents the standard deviation of the unexplained variance, wherein residuals are found by subtracting the frequency value predicted by the data model from the actual measured frequency value. Generally, RMSE is considered to be the most appropriate metric if the data model’s primary purpose is for prediction [53]. As the data models’ purpose was to predict the bridge’s future frequency/dynamic behaviour, RMSE was deemed a suitable metric.

Another important factor when assessing a model’s fit and predicting power is the similarity between the training and testing data residuals. The range of both sets of residuals needs to be comparable to maximise the data model’s predicting power. For this purpose, the interquartile range (IQR) of the residuals will also be reported for our four data models.

**Figure 11:**
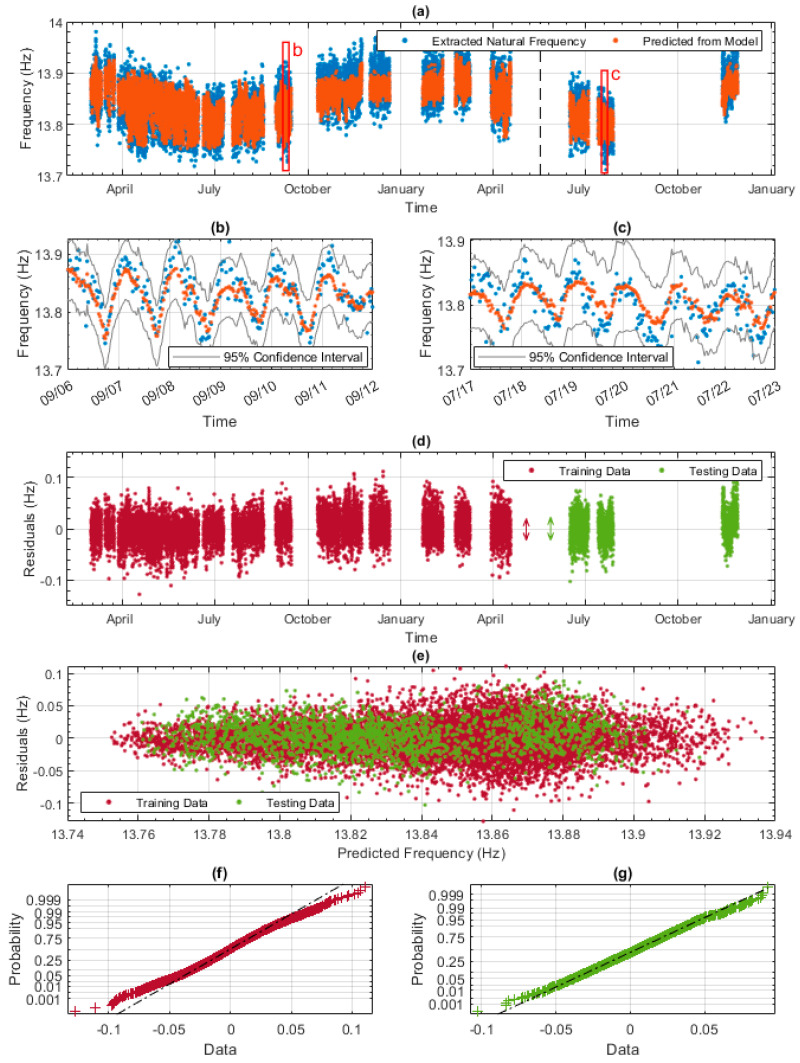
Results from the 5th-mode data model: (**a**) the full experimental data set and corresponding values predicted by the regression model (divided into training and testing), (**b**) zoomed-in view showing approximately 1 week of data during the training phase, shown in (**a**) by frame labelled b, (**c**) zoomed-in view showing approximately 1 week of data during the testing phase, shown in (**a**) by frame labelled c, (**d**) training and testing residuals across the whole monitoring period, (**e**) residuals compared to predicted frequency from the data model, (**f**) normal probability plot of the training residuals, and (**g**) normal probability plot of the testing residuals.

Figure 11a–c shows the results from the data model developed using the workflow described in Section 5.2. Gaussian Process Regression (GPR) was used and the dependent variable was the fifth natural frequency. GPR was used as it gave slightly better results than a linear regression model. In Figure 11a, the experimentally measured frequencies are shown with blue dots, and the predicted frequencies from the GPR model are shown with orange dots. The black vertical line separates the training and testing data. The seasonal variation of the natural frequency is evident in Figure 11a, which shows that the data model does an accurate and good job of predicting the increase in frequency during winter and the decrease in frequency during summer. Figure 11b,c show, in more detail, one week of training data and of testing data, respectively. Figure 11b,c show, again, that the model does a good job of predicting the daily trends. The 95% confidence intervals are also shown in both Figure 11b,c (grey lines) to indicate the level of certainty of the model. At this scale, the model occasionally underestimates the highest frequencies and overestimates the lowest frequencies. The vast majority of the experimental (blue) data points are within the 95% confidence intervals (only 5 are outside). This is particularly significant for the testing data (Figure 11c) as it shows that once trained, the model can provide very credible envelopes for expected future behaviour.

Figure 11d shows the residuals of the model against time, allowing a comparison of model performance between the training and testing data. The RMSE values of 0.0252 and 0.0265 for the training and testing data can be considered averages of the residuals. Observing Figure 11d also allows the analyst to identify whether there were any periods of significantly better or worse prediction ability. As stated previously, it is essential to limit the variation between the training and testing residuals to maximise the model’s predictive power. The variation between the training and testing residuals is measured using the interquartile range of each. The interquartile ranges of 0.0307 and 0.0344 are represented by the red and green double arrows in Figure 11d.

Figure 11e plots the residuals of the model against the predicted frequencies from the model. This figure enables the checking of any issues discussed in Section 5.2, specifically checking whether the underlying assumptions still hold. Finally, the residuals of the model are checked to see whether they are normally distributed, which is one of the regression assumptions. Figure 11f for training residuals and Figure 11g for testing residuals show normal distributions using normal probability plots. The majority of both sets of residuals fall on the dashed line, meaning they have normal distributions.

When the data for modes 1 to 3 were modelled, plots similar to Figure 8 were developed for each mode. The summary metrics from all the plots are presented in Table 4. Table 4 shows the RMSEs and interquartile ranges of both the training and testing data for all four data models, i.e., one data model for each of the four natural frequencies for which data is available. The RMSEs and interquartile ranges shown for mode 5 represent the values quoted when describing Figure 11. From the table, it can be seen that the magnitude of the RMSEs and interquartile ranges for modes 1–3 are similar to the metrics for mode 5. The range of the RMSE is from 0.0092 Hz (from the mode 1 model) to 0.0355 Hz (from the mode 3 model). These RMSE values indicate that the data models show high levels of accuracy, as the magnitude of the RMSE shows a model’s average error when predicting the natural frequency.

Table 4 also reports the IQR of the training and testing data residuals. During the data models’ training, the interquartile range values between the training and testing data were checked to ensure that they were as similar as possible. Similar interquartile range values are an indication that a data model’s predictive power has been maximised. Like for mode 5, the IQR values for the other modes were found to be similar in magnitude.

## 6. Identifying the Frequency Shift That Can Be Detected by the Data Models

The overall goal of the MID approach is the identification of structural changes/damage in short- and medium-span bridges. This section outlines how the data models were tested to determine whether it was feasible to use them to identify damage. The data models track a given natural frequency of the bridge. Using a data model to predict future behaviour allows any anomalous change in frequency to be identified. Damage to the bridge will cause a change in its natural frequency. The smaller the frequency shift that can be detected is, the more sensitive to damage a data model will be.

Through experimental and simulated processes, other studies such as [54,55,56] have identified changes in the natural frequencies of a structure due to different damage cases. The frequency shifts noted in these studies range from minor damage, causing a 0.003 Hz shift, to the loss of 73% of the prestressing load, causing a 1.79 Hz shift. These studies give indications of the size of the frequency shift that needs to be identifiable by a data model if damage is to be detected. While these studies are all different, it appears that low severity damage generates frequency shifts of the order of 0.01 Hz, with larger damage resulting in shifts of the order of 0.2 Hz.

**Figure 12:**
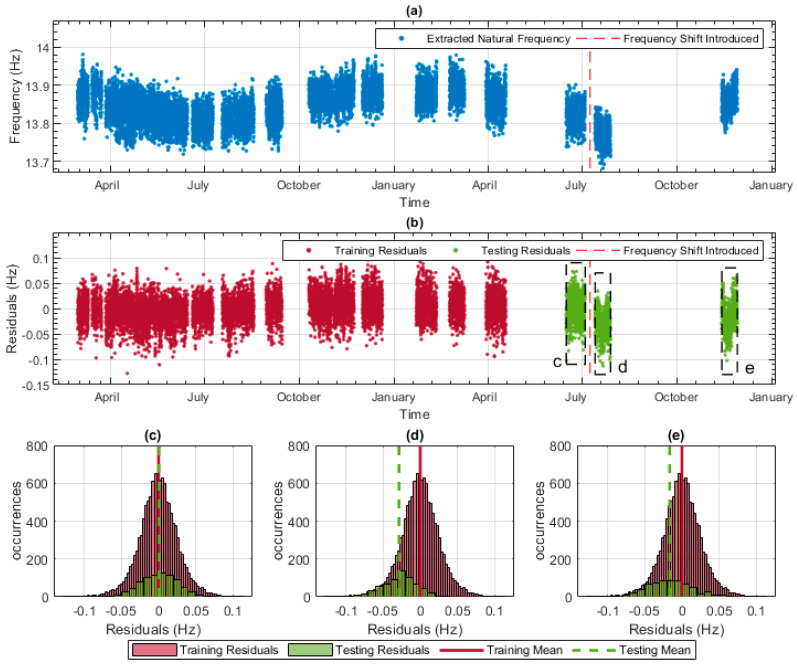
Process used to detect frequency shift: (**a**) all experimentally measured frequency data with a −0.03 Hz shift introduced to data to the right of the red dashed line, (**b**) the training and testing residuals across the whole monitoring period, (**c**) the histogram showing training residuals and testing residuals indicated by the frame labelled ‘c’ in (**b**), (**d**) the histogram showing training residuals and testing residuals indicated by the frame labelled ‘d’ in (**b**), (**e**) the histogram showing training residuals and testing residuals indicated by the frame labelled ‘e’ in (**b**).

To detect the level of frequency shift that can be identified by the four data models, the following procedure was followed:

- A small artificial frequency shift was introduced within the testing frequency data. Figure 12a plots all the frequency data and marks the point at which the frequency shift was introduced (i.e., the red dashed vertical line). The shift in frequency (0.03 Hz in this case) is not easily identifiable in Figure 12a.
- The data model for this mode was used to produce residuals for the entire monitoring period, including the frequency data with the shift applied. These residuals can be seen in Figure 12b, again with the point at which the frequency shift was introduced being indicated by the red dashed vertical line. Using this plot, the two sections of testing data, marked with the boxes ‘d’ and ‘e’, appear to have lower residuals than the training data and the testing data section with no shift applied (box ‘c’).
- To make it easier to identify a shift, the distribution of the residuals enclosed by box ‘c’, indicated in Figure 12b, has been plotted in Figure 12c, which also includes the distribution of the residuals of the training data. Figure 12c shows that the training data distribution and mean are very similar to those of section ‘c’ of the testing data, which does not have the frequency shift applied. Figure 12d,e show that the distributions of sections ‘d’ and ‘e’ of the testing data differ from those of the training data. This change in the residuals implies that the bridge’s predicted behaviour does not match what has been observed historically. Thus, the data model has correctly identified that there has been a change in frequency.
- A frequency shift of 0.02 Hz was also applied, and while a shift was evident, it was not as clear as the 0.03 Hz shift shown in Figure 12; thus, the lowest frequency shift that was reliably detectable for this mode was deemed to be 0.03 Hz.

Using the process described above, frequency shifts were also applied to modes 1–3 to determine detectable shift levels, and the results have been presented in Table 5. The first row of Table 5 shows the mean frequency of each mode. The second row shows the difference between the highest frequency observed for the mode and the lowest frequency observed for the mode. For modes 1 and 2, a frequency shift of 0.01 Hz was detectable by the data model, whereas for modes 3 and 5, a frequency shift of 0.03 Hz was detectable. Comparing the detectable frequency shifts in Table 5 to the frequency shifts due to damage that have been reported by others, the data models would be able to detect a large proportion of the damage implemented in the reviewed studies. While this is not definitive proof that the data models could detect damage in the bridge in this study, it shows that the frequency shift they can detect is of the same order of magnitude as that caused by damage, which is a significant finding.

## 7. Simulating Damage via Finite Element Modelling

In Section 3, a FE model of the studied bridge was developed, and in Section 5, it was determined that an abnormal frequency shift greater than 0.01 Hz and 0.03 Hz could be detected in the frequency data. This was comparable to or smaller than the frequency shifts reported in other studies that observed frequency shifts due to bridge damage. While the favourable comparison with other studies is encouraging, none of those bridges were identical to the bridge used in this study; therefore, it is difficult to make a direct comparison. In terms of identifying the potential usefulness of the MID approach to a bridge manager, the next step is to see what real damage these frequency shifts represented—more specifically, to examine whether the kind/severity of bridge damage that has been reported as detectable in other studies can be identified through frequency shifts of 0.01–0.03 Hz. A Finite Element (FE) model of the bridge was created, updated, and used to simulate damage in this section.

In Section 1, it was shown that a stiffness loss in the region of 25% was representative of what was being claimed to be identifiable in other studies. Therefore, a loss of stiffness of 25% in one main beam is used as a starting point for the damage simulations in this section. The damage scenarios simulated are shown in Table 6, including their locations and implementation in the FE model. The damage scenarios have been chosen to provide a range of local damage cases and more widespread global damage. Simulating both local and global damage cases will show which causes more significant changes in the bridge’s natural frequency.

**Figure 13:**
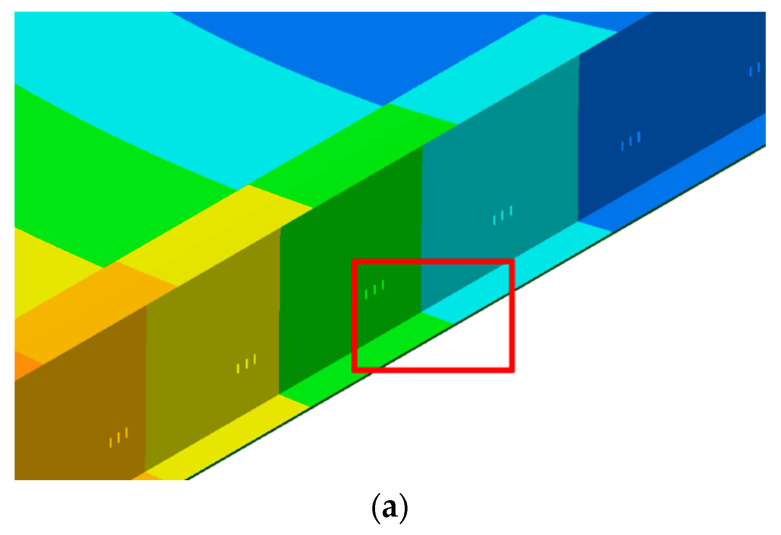

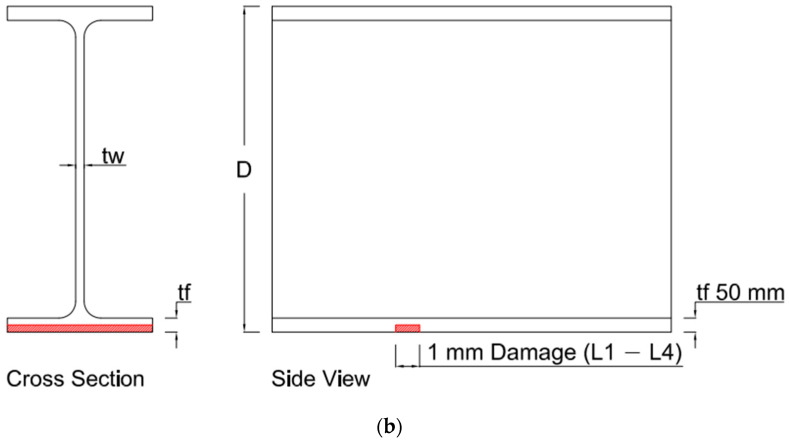
FE model damage scenarios: (**a**) location of local cut to main girder beam, shown with red frame (L1–L5); (**b**) diagram showing lossof section in damage scenarios.

The first row of Table 6 describes damage scenario L1 (Local 1), which simulates a localised loss of stiffness at the midspan of the bridge. Specifically, it simulates a reduction in the thickness of the bottom flange from 50 mm to 25 mm, and this is implemented over a distance of 1 mm in the longitudinal direction. A visual representation of the damage is shown in Figure 13. Figure 13a shows a 3D view of the girder with the location of the local damage indicated with a red box. Figure 13b shows a diagram of the cross-section and side view of the main girder, with the local damage scenarios indicated on the relevant part of the girder with a red hatch. The L1 damage is equivalent to reducing the moment of inertia of the primary beam by 23.6% over a 1 mm longitudinal length. L2–L4 represent the same level of damage at a ⅟_3_ span, ¼ span, and ⅟_5_ span, respectively. L5 is a larger damage case that reduces the moment of inertia by 50% over 1 mm. This damage case has been included as it is close to the upper end of the local damage severities reported by others. A larger local damage case is required to determine how the frequency would be affected at the upper limit of reported detectable damage.

The widespread damage case W1 simulates a global loss of section in the lower flange of one girder by reducing the flange thickness from 50 mm to 45 mm over the entire 36 m length of the bridge. This may represent such damage as uniform corrosion. This damage represents a 4.5% reduction in the moment of inertia of the beam. Widespread damage case W2 simulates a global loss of section in the web of one girder by reducing web thickness from 20 mm to 19 mm over the 36 m length of the bridge. This damage represents a 1.7% reduction in the moment of inertia of the beam.

The results from all damage simulations are presented in Table 7. Columns 2–5 in the table show the frequency shift caused by each damage case for modes 1, 2, 3, and 5, respectively. For reference, the mean frequency for each of the (healthy) modes is also shown in the first row of the table.

In Section 5, it was established that the developed data models could detect frequency shifts of 0.01 Hz, 0.01 Hz, 0.03 Hz, and 0.03 Hz in modes 1, 2, 3, and 5, respectively. Here, the data models that could detect the frequency shift are highlighted in green. Several frequency shifts are almost in the detectable range (within 20%) and are highlighted in orange. The data models could detect the smallest simulated damage at 3 of the 4 tested locations (L2 to L4). The smallest local damage located at the mid-span of the bridge (L1) did not produce a frequency shift large enough to be detected by the data models. Both of the widespread damage scenarios could be detected by the data models. The 1 mm loss of section to the web (W2) was detectable by all the data models, while the 5 mm loss of section to the flange (W1) was only detectable by the first frequency data model.

Using the results presented in Table 7, it is possible to make the following observations beyond what damage is detectable and not detectable:

- Each of the four data models had a different sensitivity to each of the damage cases. The fifth frequency data model could detect damage cases L2, L3, L4, and W2, while the first frequency data model could detect damage cases L5, W1, and W2. This result indicates that all frequencies should be monitored to increase the likelihood of one of them detecting damage.
- The same type of local damage located at different positions on the bridge span results in different frequency shifts. This shows that both the magnitude and location of damage should be considered when investigating what level of damage an SHM system can detect.
- Modes 3 and 5 seem to have higher sensitivities to damage. The average frequency shifts caused by the damage for L1 to L4 (same size of damage, differing locations) are 0.016 Hz and 0.032 Hz for frequencies 3 and 4, respectively. The same average damage shifts for frequencies 1 and 2 are only 0.003 Hz and 0.005 Hz, respectively. This finding agrees with various other studies [57,58] that have shown that higher-order modes are more sensitive to damage.
- Based on the literature [59], higher modes tend to be better at identifying damage due to their being generally larger in magnitude. However, modes 3 and 5 were both unable to identify the localised stiffness loss at mid-span (damage L1). Initially, this seems surprising. One might think that it should be able to identify damage at mid-span if it can detect it at the 1/3, ¼, and 1/5 points. However, mid-span is a node point for modes 3 and 5 (see Figure 2), and so, a local stiffness loss there has a relatively small effect on the frequencies of these modes. It is unfortunate that the position of the sensor enclosure meant that mode 4 was not picked up in the long-term data as the anti-node for mode 4 is located at mid-span (Figure 2), and it is likely that it would have been able to detect L1.

## 8. Conclusions

The MID approach presented in this work utilises low-cost sensing equipment, making it potentially feasible for operators to implement across a network and monitor multiple smaller bridges simultaneously. This paper aimed to investigate whether this new low-cost MID approach could provide valuable information for bridge management. Specifically, it sought to determine the detectable frequency shift level and assess whether it was small enough to identify the types of damage reported in the existing literature.

To evaluate the MID approach, acceleration and temperature data were collected from a 36 m span steel bridge over a period of approximately 2 years. Our study incorporated selected elements of modelling best practices from other domains to develop a customised data modelling workflow specifically suited for sparsely instrumented short- and medium-span bridges. While the individual elements of the method are not new, their integration within a workflow tailored for sparsely instrumented bridges represents an important development.

The study found that the MID approach was capable of detecting a frequency shift as small as 0.01 Hz, a magnitude associated with various damages reported in the literature. This suggests that the MID approach has the potential to provide valuable information for bridge management.

Furthermore, a finite element (FE) model of the bridge was created to assess the MID approach’s ability to identify the level of damage in the presence of confounding influences. A localized stiffness loss of 25%, which is typically considered detectable in the literature under limited confounding effects, was simulated. The study considered this level of stiffness loss to be at the lower end of what is currently claimed as detectable when confounding effects such as temperature are present. The results showed that six out of the seven simulated damage scenarios could be detected by the MID data models. This included both the detection of a reduction in the primary beam moment of inertia by 23.6% (for 1 mm longitudinally) in most cases as well as the detection of widespread damage equivalent to a moment of inertia as low as 1.7% across the main girder. However, it is important to note that in real-world cases, the detectability of localised damage may vary depending on the specific points on a bridge.

The main advantage of using the MID method over other data modelling approaches is that it has been developed to extract useful bridge health metrics from sparse data. Whereas other methods can pick and choose which sensor outputs will be used as inputs for their data models, in MID, the limited data means that the choice and placement of the sensors are more critical than in other data modelling approaches.

In summary, the paper’s contributions lie in the development of a customized data modelling workflow for sparsely instrumented bridges and the successful detection of abnormal frequency shifts and comparable damage levels using the MID approach. The use of low-cost sensing equipment and the potential for the simultaneous monitoring of multiple bridges enhance the practicality of the MID approach and make it a viable option for network-level bridge monitoring.

## Figures and Tables

**Table 1:** Material properties used in FE model.

	Steel	Concrete
Young’s modulus	206 × 10^6^ kN/m^2^	30 × 10^6^ kN/m^2^
Density	7800 kg/m^3^	2550 kg/m^3^
Poisson’s ratio	0.3	0.2

**Table 2:** Modal property comparison between FE model calibration and measurement.

	Frequency Reference				
	1st Mode	2nd Mode	3rd Mode	4th Mode	5th Mode
Modal Test	3.08	4.94	7.48	11.34	13.78
FE model	3.01	4.55	7.49	11.38	13.92
Difference between FE model and modal test (Hz)	0.07	0.39	0.01	0.04	0.14
Difference (%)	2.27	7.89	−0.13	−0.35	−1.02

**Table 3:** Statistics of the identified natural frequencies.

Average Frequency (Hz)	Frequency Range (Hz) (After Basic Outlier Removal)	Relative Variation (%)
3.0963	0.0739	2.39
4.9497	0.0971	1.96
7.5550	0.3250	4.30
13.8452	0.2834	2.05

**Table 4:** RMSE and interquartile range values for all data models.

	Mode 1		Mode 2		Mode 3		Mode 5	
	Training	Testing	Training	Testing	Training	Testing	Training	Testing
RMSE (Hz)	0.0092	0.0094	0.0099	0.0105	0.0337	0.0355	0.0252	0.0265
IQR of the Residuals (Hz)	0.0108	0.0113	0.0120	0.0136	0.0417	0.0431	0.0307	0.0344

**Table 5:** Frequency mean range and frequency that can be detectable by the data models.

	Mode 1	Mode 2	Mode 3	Mode 5
Mean frequency for the mode (Hz)	3.0963	4.9497	7.5550	13.8452
Range between highest and lowest frequency observed for the mode (Hz)	0.0739	0.0971	0.3250	0.2834
Frequency shift that was Detectable (Hz)	0.01	0.01	0.03	0.03

**Table 6:** Simulated damage scenarios.

Reference	Description of Damage	Location of Damage	Simulation Method in FE
L1	1 × 632 × 25 mm transverse cut to bottom flange	½ span	Reduction of plate thickness at damage location
L2	1 × 632 × 25 mm transverse cut to bottom flange	⅟_3_ span	Reduction of plate thickness at damage location
L3	1 × 632 × 25 mm transverse cut to bottom flange	⅟_4_ span	Reduction of plate thickness at damage location
L4	1 × 632 × 25 mm transverse cut to bottom flange	⅟_5_ span	Reduction of plate thickness at damage location
L5	1 × 632 × 50 mm transverse cut to bottom flange	½ span	Reduction of plate thickness at damage location
W1	Loss of 5 mm from the bottom flange of one main girder	Southern main girder	Reduction of plate thickness at damage location
W2	Loss of 1 mm from the web of one main girder	Southern main girder	Reduction of plate thickness at damage location

**Table 7:** Changes in natural frequency caused by each of the damage scenarios.

		Frequency Reference *			
		F1	F2	F3	F5
	No Damage Mean Frequency (Hz)	3.08	4.94	7.48	13.78
Frequency Shift from mean (Hz)	L1: 23.6% loss of stiffness at ½ span	0.0067	0.0097	−0.0026	−0.0006
	L2: 23.6% loss of stiffness at ⅟_3_ span	0.0032	0.0052	0.0198	0.0496
	L3: 23.6% loss of stiffness at ⅟_4_ span	0.0011	0.0031	0.0243	0.0463
	L4: 23.6% loss of stiffness at ⅟_5_ span	0.0008	0.0030	0.0215	0.0331
	L5: 50% loss of stiffness at ½ span	0.0202	0.0277	−0.0012	0.0034
	W1: 4.5% loss of stiffness over whole flange	0.0102	0.0051	0.0229	0.0265
	W2: 1.7% loss of stiffness over whole web	0.0167	0.0163	0.0302	0.0581

* green background: data model could detect the frequency shift, orange background: almost in the detectable range of data model (within 20%).

## Data Availability

Some or all data, models, or code that support the findings of this study are available from the corresponding author upon reasonable request.
